# External validation of a modified cardiovascular sequential organ failure assessment score in patients with suspected infection using the MIMIC-IV database

**DOI:** 10.1371/journal.pone.0312185

**Published:** 2024-11-12

**Authors:** Sung Yeon Hwang, Inkyu Kim, Byuk Sung Ko, Seung Mok Ryoo, Eunah Han, Hui Jai Lee, Daun Jeong, Tae Gun Shin, Kyuseok Kim, on behalf of the Korean Shock Society

**Affiliations:** 1 Department of Emergency Medicine, Samsung Medical Center, Sungkyunkwan University School of Medicine, Seoul, Korea; 2 Department of Digital Health, Samsung Advanced Institute for Health Sciences & Technology, Sungkyunkwan University, Seoul, Korea; 3 Department of Emergency Medicine, College of Medicine, Hanyang University, Seoul, Korea; 4 Department of Emergency Medicine, Asan Medical Center, University of Ulsan College of Medicine, Seoul, Korea; 5 Department of Emergency Medicine, Gangnam Severance Hospital, Yonsei University College of Medicine, Seoul, Korea; 6 Department of Emergency Medicine, SMG-SNU Boramae Medical Center, Seoul, Korea; 7 Department of Emergency Medicine, Seoul National University College of Medicine, Seoul, Korea; 8 Department of Emergency Medicine, CHA Bundang Medical Center, CHA University School of Medicine, Seongnam, Korea; Mahidol University, Faculty of Tropical Medicine, THAILAND

## Abstract

We developed a modified cardiovascular (CV) Sequential Organ Failure Assessment (SOFA) score using an emergency department-based cohort data, incorporating norepinephrine equivalent dose and lactate to represent current clinical practice patterns for vasopressor utilization and the diagnostic significance of lactate, respectively. In this study, we sought to validate this modified CV-SOFA score in intensive care unit patients with suspected infection using the Marketplace for Medical Information in Intensive Care (MIMIC)-IV database. This was a retrospective study that utilized data from the MIMIC-IV database. Modified CV/total SOFA score and original CV/total SOFA score were compared for predicting in-hospital mortality. Area under the receiver operating characteristic curve (AUROC) and the calibration curve were employed to evaluate discrimination and calibration, respectively. A total of 29,618 ICU patients with suspected infections was analyzed. The in-hospital mortality rate was 12.4% (n = 3,675). Modified CV-SOFA score (AUROC 0.667; 95% confidence interval [CI] 0.657–0.677 vs. 0.663; 95% CI 0.654–0.673; p = 0.283) and modified total SOFA score (0.784 [95% CI 0.776–0.793] vs. 0.785 [95% CI 0.777–0.793], p = 0.490) did not differ significantly from the original CV-SOFA score and original total SOFA score, respectively. The calibration curve of the original CV-SOFA score was inferior to that of the modified CV-SOFA score. The modified CV- and total SOFA scores were better calibrated than the original CV- and total SOFA scores, but their discriminative performance was not significantly different. Further studies of the modified CV-SOFA score in different settings and populations are required to assess the generalizability of this score.

## Introduction

Organ failure is the leading cause of mortality and morbidity among patients hospitalized in the intensive care unit (ICU) [1, 2]. The use of severity scoring tools in ICU patients can help to improve clinical decision-making, allocate resources appropriately, standardize research practices, and develop benchmarks for quality of patient care across ICUs [3]. The Sequential Organ Failure Assessment (SOFA) score has become one of the most extensively used scoring systems in the field of critical care since its introduction [4]. The primary objective of the SOFA score is to quantify and objectively evaluate the degree of organ dysfunction in critically ill patients over time. However, the SOFA score has been validated in several other contexts, such as prognostication in a range of ICU settings and as a surrogate endpoint for mortality in clinical trials [5–7]. In addition, the SOFA score was utilized in the most recent version of the definition of sepsis in patients with suspected infections [8].

The SOFA score was established during a time when dopamine was the preferred vasopressor and when vasopressor use was more conservative than it is currently. In the past decade, norepinephrine has surpassed dopamine as the favored vasopressor in patients with circulatory shock [9]. The administration of vasopressors is shifting toward more liberal and earlier administration without large volumes of resuscitation fluids, and dobutamine is rarely used alone [10, 11]. Furthermore, other vasopressors, such as vasopressin, which is now commonly used, and numerous mechanical circulatory support devices such as extracorporeal membrane oxygenation machines, have been widely used in the past decade but are not represented in the SOFA score [12, 13]. Therefore, implementing the CV-SOFA score in its current form may not adequately reflect the severity of organ failure. Thus, the scoring system must be updated to reflect contemporary practice and patient demographics [14].

Recently, we proposed a modified CV-SOFA score incorporating norepinephrine equivalent dose and lactate to represent current clinical practice patterns for vasopressor utilization and the diagnostic significance of lactate, respectively (Table 1) [15]. Its validity was assessed both internally and externally, and it outperformed the original system in a cohort of patients with suspected infections, sepsis, and septic shock. Concerns have been expressed, however, regarding the generalizability of the modified CV-SOFA score, given that its development and validation were limited to an emergency department-based cohort. In this investigation, we sought to validate a modified CV-SOFA score in ICU patients with suspected infection using the Marketplace for Medical Information in Intensive Care (MIMIC)-IV database.

**Table 1:** Original and modified cardiovascular SOFA scores.

Score	Original cardiovascular SOFA	Modified cardiovascular SOFA (M3)
0	MAP ≥70 mmHg	MAP ≥70 mmHg
		Add 1 point if lactate ≥2 mmol/L
1	MAP <70 mmHg	MAP <70 mmHg *OR* NEq ≤0.2
		Add 1 point if lactate ≥2 mmol/L
2	Dopamine ≤5	0.2< NEq ≤0.5
	Dobutamine (any dose)	Add 1 point if lactate ≥2 mmol/L
3	Dopamine >5 *OR*	NEq >0.5
	epinephrine ≤0.1 OR norepinephrine ≤0.1	Add 1 point if lactate ≥2 mmol/L
4	Dopamine >15 *OR*	NEq >0.5 *AND*
	epinephrine >0.1 *OR* norepinephrine >0.1	Lactate ≥2 mmol/L

Vasopressor doses are given as μg/kg/min at least 1 h.

*SOFA* Sequential Organ Failure Assessment, *MAP* mean arterial pressure, *NEq* norepinephrine equivalent dose

## Methods

### Study design and data source

A retrospective cohort analysis was conducted using data from the MIMIC-IV database, which contains an extensive amount of anonymized clinical data. This database contains data from patients admitted to Beth Israel Deaconess Medical Center in Boston, MA, United States, between 2008 and 2019.

The researcher has completed "Protecting Human Research Participants" training and possessed the qualifications necessary to utilize the database and extract pertinent data at the time of the study. We accessed the MIMIC-IV database on 27/10/2021. Using PostgreSQL tools version 10.16, created by the PostgreSQL Global Development Group in California, and Python version 3.7, data extraction and manipulation procedures were executed. Age, gender, co-morbidities, type of intensive care unit (ICU), vital signs, laboratory results from the first day, Glasgow coma scale, vasoactive drugs, organ support interventions including mechanical ventilation and renal replacement therapy, and mortality rates were abstracted. For patients with multiple ICU admissions, only the first was considered.

The data contained within this database have been deidentified to remove any patient-specific information. This study was approved by the institutional review boards of Samsung Medical Center (No. 2021–09‑034). All methodologies utilized in this study were conducted in accordance with applicable rules and regulations.

### Study population

Patients who met the following criteria were included in the study: 1) admitted to the ICU and older than 18 years at the time of admission and 2) exhibited evidence of infection suspicion on the initial day of ICU admission. Suspicion of infection was defined as the presence of all of the following conditions [16]: 1) body fluid cultures collected within a 24-hour timeframe after the administration of antibiotics and 2) antibiotics given within a 72-hour window following body fluid cultures, with the prescription of antibiotics on the first day of admission to the ICU.

### Calculation of the SOFA score

The original SOFA score was calculated by selecting the most severe measurements available for each variable within the first 24 hours following ICU admission. Consistent with the methodology employed in previous studies, we assigned a score of zero to all variables with missing data, assuming their normality. To calculate the SOFA score, laboratory tests performed from 6 hours prior to ICU admission to 24 hours after admission were utilized. In situations where multiple values were captured, only the highest value was used. The CV-SOFA score is based on the administration of vasoactive drugs for a continuous duration of more than 1 hour within a 24-hour window following ICU admission. In instances where multiple drugs were administered for more than 1 hour, the drug with the highest SOFA score was used to calculate the overall score.

The development and validation processes of the modified CV-SOFA score have been described in detail [15]. In brief, several potential models incorporating different blood pressure cut-off values, vasopressor doses, and cut-off lactate levels were evaluated for the purpose of developing a modified CV-SOFA score. Hypotension was defined as a mean arterial blood pressure of 70 mmHg or less. The doses of vasopressors other than norepinephrine were converted to norepinephrine equivalent (NEq) doses (S1 Table in S1 File) [17]. When the serum lactate level exceeded a certain threshold, which is referred to as the lactate criterion, and the modified CV-SOFA score was between 0 and 3 points, an additional point was added to the modified CV-SOFA score. The model used lactate data from 6 hours prior to ICU admission to 24 hours after ICU admission. In a previous validation study, the M3 model showed the best discrimination, calibration, incidence, and mortality rate prediction (S2 and S3 Tables in S1 File) [15]. In this model, the following criteria were used to assign points: 1 point for MAP 70 mmHg or NEq ≤ 0.2 μg/kg/min; 2 points for NEq > 0.2 μg/kg/min and ≤ 0.5 μg/kg/min; 3 points for NEq >0.5 μg/kg/min; and 4 points for NEq > 0.5 μg/kg/min and lactate levels of 2 mmol/L or more. The lactate threshold was 2 mmol/L or more.

### Primary outcome

The primary outcome was in-hospital mortality.

### Statistical analysis

Continuous data are presented as means (standard deviations, SD) or medians (interquartile ranges, IQRs) and compared with Student’s t-test or Wilcoxon rank sum test, as applicable. Categorical data are presented as frequencies (percentages), and the chi-square test was used to compare data between groups. AUROC and calibration curves were used to evaluate the discrimination and calibration of the original CV/total SOFA score and the modified CV/total SOFA score for predicting in-hospital mortality.

Statistical significance was defined as a two-tailed p value of less than 0.05. All analyses were conducted using R version 3.6.3 (R Foundation for Statistical Computing, Vienna, Austria) and STATA version 17.0 (STATA Corporation, College Station, TX).

## Results

### Baseline characteristics

A total of 29,618 patients was included in the primary analysis (S1 Fig in S1 File). Baseline characteristics of the study cohort are shown in Table 2. Overall, in-hospital mortality was 12.4% (n = 3,675). The study cohort had a mean age of 65.4 (SD 16.7) years, and 56.7% were male (non-survivor group vs. survivor group: 53.7% vs. 57.2%, p<0.001). Mean Charlson comorbidity score was significantly higher in non-survivors than survivors (7.0 ± 3.0 vs. 5.3 ± 2.9, p <0.001). Vasopressors were given to 34.3% of the patients, and their frequency of use was significantly higher in non-survivors than in survivors (54.6% vs. 31.1%, p<0.001). Vasopressor dosage, as measured by NEq dose, was significantly higher in the non-survivor group (0.40 μg/kg/min [0.37] vs. 0.15 μg/kg/min [0.19], p<0.001). Overall, mean SOFA score was 5.2 (3.5), and the non-survivor group had substantially higher SOFA scores than the survivor group (8.5 [4.4] vs. 4.7 [3.1], p<0.001).

**Table 2:** Baseline characteristics.

Variables	All patients	Survivors	In-hospital Death (n = 3,675)	p
	(n = 29,618)	(n = 25,943)		
Age (mean ± SD), years	65.4 ± 16.7	64.7 ± 16.7	69.8 ± 15.6	<0.001
Male sex, No. (%)	16,806 (56.7)	14,833 (57.2)	1,973 (53.7)	<0.001
Charlson comorbidity score (mean ± SD)	5.5 ± 2.9	5.3 ± 2.9	7.0 ± 3.0	
Intensive care unit				<0.001
MICU	6,532 (22.1)	5,393 (20.8)	1,139 (31.0)	
MICU/SICU mixed	5,787 (19.5)	4,836 (18.6)	951 (25.9)	
CCU	2,590 (8.7)	2,138 (8.2)	452 (12.3)	
SICU/CV-ICU	14,399 (48.6)	13,282 (51.2)	1.117 (30.4)	
Neuro ICU	310 (1.1)	294 (1.1)	16 (0.4)	
Vital signs^a^				
Lowest MAP (mean ± SD), mmHg	61.5 ± 11.5	62.3 ± 11.0	55.8 ± 13.6	<0.001
Highest HR (mean ± SD), beat per min	105.0 ± 20.6	103.9 ± 19.9	113.2 ± 23.4	<0.001
Highest RR (mean ± SD), breath per min	28.4 ± 6.6	28.1 ± 6.5	31.0 ± 7.2	<0.001
Highest Temperature, °C	37.4 ± 0.8	37.5 ± 0.8	37.3 ± 1.1	<0.001
Laboratory findings^a^ (mean ± SD)				
Highest WBC, 10^9^/L	15.3 ± 11.9	14.9 ± 10.8	18.1 ± 17.4	<0.001
Lowest Hb, g/dl	9.6 ± 2.4	9.6 ± 2.3	9.9 ± 2.7	<0.001
Lowest platelet, 10^9^/L	171.8 ± 81.3	174.0 ± 79.2	155.8 ± 93.0	<0.001
Highest bilirubin, mg/dL	2.1 ± 4.7	1.8 ± 3.8	3.7 ± 7.4	<0.001
Highest creatinine, mg/dL	1.5 ± 1.5	1.4 ± 1.5	2.1 ± 1.7	<0.001
Highest Lactate, mmol/L	2.9 ± 2.5	2.6 ± 1.8	4.9 ±4.4	<0.001
GCS score^a^ (mean ± SD)	13.5 ± 2.9	13.6 ± 2.8	12.8 ± 3.6	<0.001
Vasopressor use^a^, No. (%)`	10,156 (34.3)	8,151 (31.4)	2,005 (54.6)	<0.001
Norepinephrine, No. (%)	5,392 (18.2)	3,779 (14.6)	1,613 (43.9)	<0.001
Epinephrine, No. (%)	1,489 (5.0)	1,186 (4.6)	303 (8.2)	<0.001
Dopamine, No. (%)	601 (2.0)	368 (1.4)	233 (6.3)	<0.001
Vasopressin, No. (%)	1,611 (5.4)	845 (3.3)	766 (20.8)	<0.001
Phenylephrine, No. (%)	5,294 (17.9)	4,505 (17.4)	789 (21.5)	<0.001
Dobutamine use, No. (%)	276 (0.9)	165 (0.6)	111 (3.0)	<0.001
Norepinephrine equivalent dose^b^ (mean ± SD), μg/kg/min	0.20 ± 0.25	0.15 ± 0.19	0.40 ± 0.37	<0.001
Mechanical ventilation^a^, No. (%)	14,812 (50.0)	12,590 (48.5)	2,222 (60.5)	<0.001
Renal replacement therapy^a^, No. (%)	629 (2.2)	420 (1.6)	209 (5.7)	<0.001
SOFA score^a^ (mean ± SD)	5.2 ± 3.5	4.7 ± 3.1	8.5 ± 4.4	<0.001

^a^The worst value and intervention on the first day of intensive care unit admission were extracted.

^b^Norepinephrine equivalent doses are given as μg/kg/min at least 1 h.

### Incidence and in-hospital mortality according to original vs. modified CV-SOFA score

**Fig 1:**
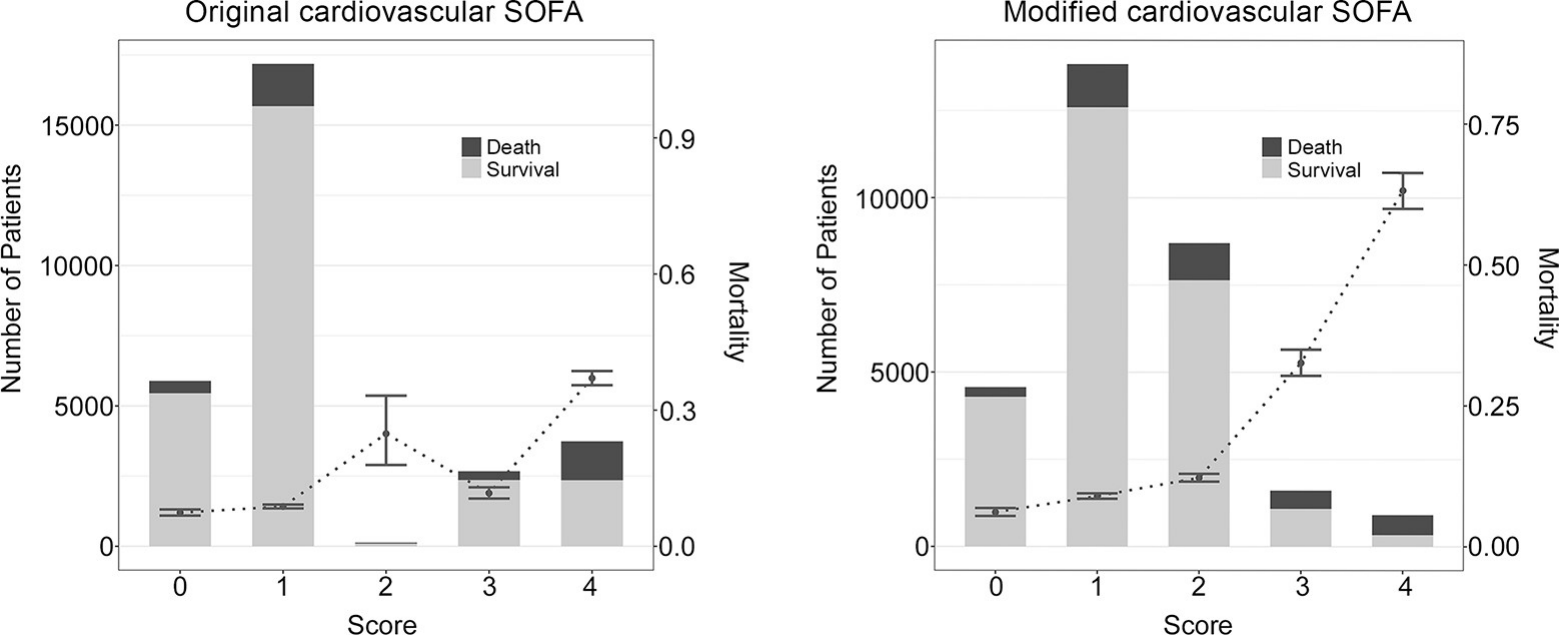
Distribution and in-hospital mortality rate according to original and modified cardiovascular/total SOFA scores. Bar graphs represent the number of patients, and points with error bars indicate 28-day mortality with 95% confidence intervals. Abbreviations: SOFA, sequential organ failure assessment.

Distribution and in-hospital mortality rate according to original and modified cardiovascular/total SOFA scores are presented in Fig 1 (also see S4 Table and S2 Fig in S1 File). The distribution of the original CV-SOFA score was skewed, as only 133 (0.5% of the patients) patients had a CV-SOFA score of 2. The in-hospital mortality rate of patients with a CV-SOFA score of 2 was higher than that of patients with a CV-SOFA score of 3 (24.8% vs. 11.7%). In-hospital mortality showed a tendency to increase as the modified CV SOFA score increased.

### Discrimination and calibration of the original vs. modified CV-SOFA score

**Fig 2:**
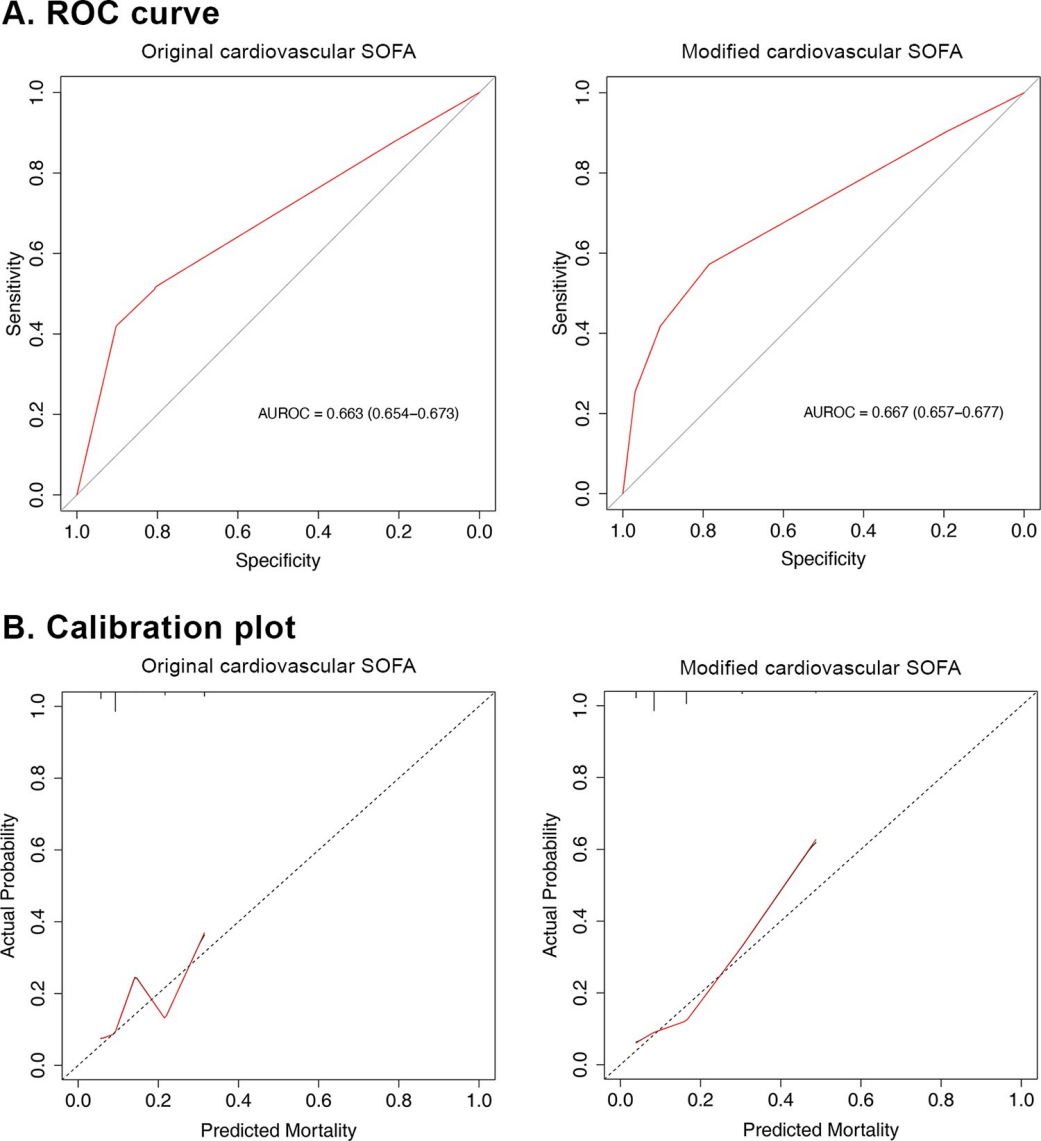
Receiver operating characteristic curves (A) and calibration plots (B) to determine the ability of the original and modified cardiovascular SOFA scores to predict in-hospital mortality.

The AUROC of the modified CV-SOFA score (0.667; 95% confidence interval [CI], 0.657–0.677) for predicting in-hospital mortality did not differ significantly from that of the original CV-SOFA score (0.663; 95% CI, 0.654–0.673; p = 0.283) (Fig 2 and S5 Table in S1 File). In addition, the AUROC of the modified total SOFA score for in-hospital mortality was not significantly different from that of the original total SOFA score (0.784 [95% CI 0.776–0.793] vs. 0.785 [95% CI 0.777–0.793], p = 0.490).

The calibration curve of the original CV-SOFA score demonstrated poor calibration (Fig 2 and S3 Fig in S1 File). On the contrary, the calibration curve for the modified CV-SOFA score model was better.

### Other modified CV-SOFA score candidates

**Fig 3:**
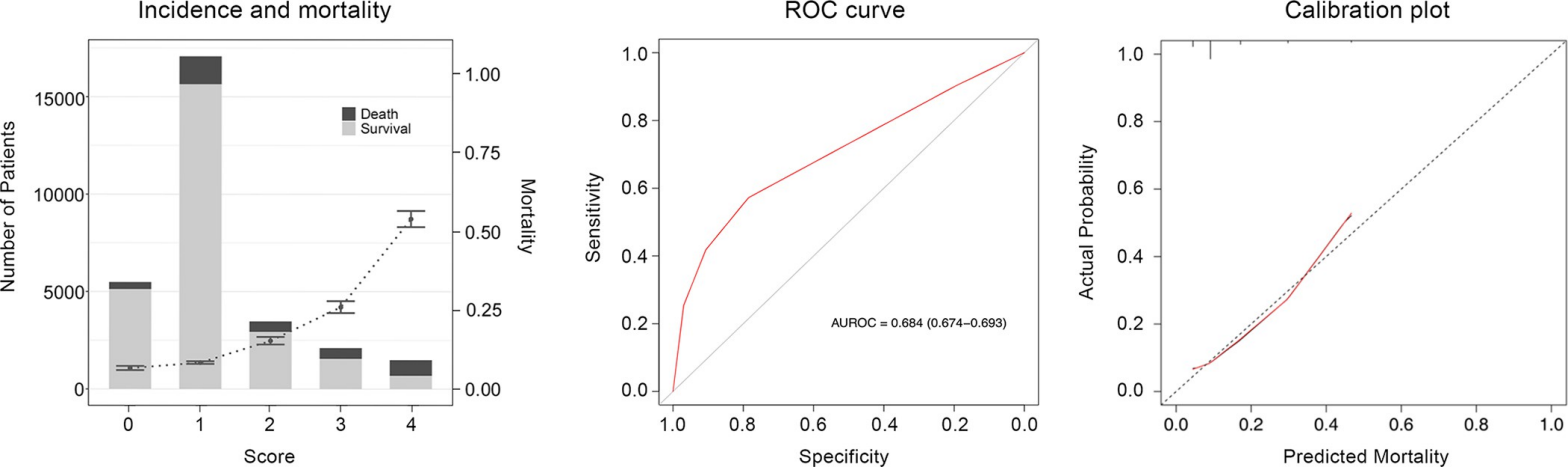
Distribution and in-hospital mortality rate (A), receiver operating characteristic curves (B), and calibration plots (C) of the M2 model.

We evaluated a model (M2) that showed the best predictive validity in the study (S5 Table in S1 File). In the M2 model, the following criteria were used to assign points: 1 point for MAP 70 mmHg or NEq ≤ 0.1 μg/kg/min; 2 points for NEq > 0.1 μg/kg/min and ≤ 0.2 μg/kg/min; 3 points for NEq >0.2 μg/kg/min; 4 points for NEq > 0.2 μg/kg/min and a lactate level of 4 mmol/L or more (S2 Table in S1 File). The lactate threshold, which was ≥4 mmol/L, is higher than that of the M3 model. The M2 model showed a similar distribution of CV-SOFA scores, but the increase in mortality was modest compared with that of the modified CV-SOFA (M3) (Fig 3). Both the CV-SOFA based on the M2 model (0.684 [95% CI, 0.674–0.693] and the modified total SOFA score (0.790 [95% CI, 0.782–0.798] demonstrated significant improvements in discrimination compared to the original SOFA score (p<0.001). In addition, the calibration curve for the modified CV-SOFA score based on M2 showed relatively better calibration than that for the modified CV-SOFA score based on M3.

## Discussion

In this study, we aimed to validate a modified CV-SOFA score that reflects current practice and to investigate if the modified CV-SOFA score showed superior predictive performance to the original SOFA score for in-hospital mortality in patients admitted to the ICU with a suspected infection using the MIMIC-IV database. The modified CV- and total SOFA scores demonstrated better calibration than the original model, but their discriminative performance did not differ significantly from that of the original scores. However, the modified CV-SOFA/total SOFA scores based on model 2 with cut-off values of lactate levels of 4 mmol/L or more and lower NEq doses (0.1 μg/kg/min to 0.2 μg/kg/min) showed significant improvement in discrimination and calibration compared to the original CV/total SOFA scores.

The modified CV-SOFA score used in this study was developed and validated primarily in a cohort of patients presenting with suspected infection in the ED [15]. Additionally, the score was validated in patients primarily in medical ICUs in Korea [18]. In this study, we used MIMIC-IV data, including data from heterogenetic patients from diverse backgrounds, all of whom were admitted to various types of ICUs in a single hospital in the United States. In a prior study, we evaluated the efficacy of the original SOFA score in patients using MIMIC-IV data and found that the SOFA score showed significant variation in prognostic value depending on the specific type of ICU. The results of that study suggested that the ICU type may be a significant prognostic adjustment factor when using the SOFA score [19]. In our study, the proportion of patients in the surgical ICU was relatively high, and the characteristics and severity of suspected infection cases in the surgical ICU may differ significantly from those in the medical ICU. In addition, patients in the MIMIC database had a lower mortality rate than in previous cohorts, and the SOFA scores of patients in the MIMIC dataset were lower. The different severity and characteristics of patients evaluated across studies could affect the optimal cut-off values of lactate and vasopressor dose for predicting mortality and the performance of the modified CV-SOFA scoring system.

A number of modifications has been proposed to the SOFA score including removing, adding, or substituting sub-components with more convenient variables [20–22]. However, few studies have attempted to address the weaknesses of the CV-SOFA score. Yadav et al. developed and validated a modified CV-SOFA score based on data from 16,386 ICU patients [23]. Their score includes all vasoactive drugs currently used in clinical practice, the shock index as a substitute for mean arterial pressure, and blood lactate as a biomarker for shock state. The modified CV-SOFA score outperformed the original version in predicting in-ICU mortality (AUC 0.801 vs. 0.718; p<0.001), in-hospital mortality (AUC 0.783 vs. 0.651; p<0.001), and 28-day mortality (AUC 0.737 vs. 0.655; p<0.001). When combined with the total SOFA score, the modified model had a higher level of predictive accuracy. Though that study included blood lactate concentrations with two cut-off values (2 mmol/L and 4 mmol/L) and commonly used vasopressors, the shock index was utilized rather than MAP.

Several studies have consistently reported that the distribution of CV-SOFA scores is biased, including few patients with a SOFA score of 2, reflecting current practice in which dopamine and dobutamine use has been drastically reduced [15, 24, 25]. Furthermore, despite the administration of a minimal quantity of norepinephrine in cases where the patient’s condition is relatively less critical, a SOFA score of 3 or 4 is assigned, resulting in overestimation of organ failure. This could adversely affect accurate determination of a patient’s prognosis and assessment of the severity of organ failure. In the study of 63,756 ICU patients conducted by Polkki et al., only 1.1% had a cardiovascular component score of 2 [24]. In addition, they demonstrated a clear increase in mortality only in patients with a CV-SOFA score of 4; they also reported that CV-SOFA scores were not associated with mortality risk, which is not comparable to other components of the SOFA score. Findings in our study were comparable and suggest that the CV-SOFA scoring system should be updated.

Our study had some noteworthy strengths. First, the overall sample size of 29,618 ICU patients from various ICU subtypes is large and included clinically diverse patients with different critical care practice patterns, enhancing the generalizability of our findings. Second, data were automatically extracted using previously established data retrieval techniques. This automation enabled large-scale data retrieval while also significantly lowering the risk of transcription error. Third, the modified CV-SOFA score covered the vast majority of commonly administered vasoactive medications and represents the current practice of administering vasopressors at low doses from the beginning of resuscitation.

In light of the varying performances exhibited by the modified models across diverse clinical settings, including emergency departments, various ICUs, and different geographical contexts such as Korea and the United States, the subsequent steps toward refining the CV or total SOFA score present considerable complexity. We propose that global collaborative efforts represent a crucial initial step in these efforts. Comprehensive datasets from multiple countries and clinical settings should be collected, and innovative methodologies, including artificial intelligence, should be employed to develop optimal predictive models.

### Limitations

This study had several limitations that should be considered. First, because this was a retrospective observational study, there is the potential for biases associated with the study design. Second, we relied on electronic health records from routine clinical practice. Since SOFA scores were derived directly from pre-existing programming languages, missing data during the calculation method may have resulted in deviations from the true value. Third, because this study population comprised patients from a single database in the United States, the results should be extrapolated with caution to other populations and regions. Fourth, some vasopressor agents, such as terlipressin and angiotensin II, were not considered. Consequently, the performance of the modified CV-SOFA score may be weakened when these vasoconstrictors are administered. Fifth, the MIMIC-IV database contains data collected over a 10-year period. As a result, our findings could have been influenced by changes in clinical practice for sepsis that occurred throughout this time period, which could have influenced the outcomes. Finally, the SOFA score was not developed to predict mortality. In the definition of Sepsis-3, however, the SOFA score was shown to be able to predict in-hospital mortality.

## Conclusions

While the modified CV/total SOFA score exhibited better calibration performance than the original, there was no significant difference in discriminative performance. To update the CV-SOFA score, further studies are needed in different settings and populations.

## Supporting information

S1 File (DOCX)
