# Supplementary material for: External validation of a modified cardiovascular sequential organ failure assessment score in patients with suspected infection using the MIMIC-IV database
Source: PLoS One. 2024 Nov 12;19(11):e0312185. doi: 10.1371/journal.pone.0312185 (PMC11556716; doi:10.1371/journal.pone.0312185)

**Supporting information**

**S1 Table.** Conversion table to determine norepinephrine equivalent dose.

**S2 Table.** Candidate models for modified cardiovascular SOFA score.

**S3 Table.** Candidate models for vasopressor only cardiovascular SOFA score

**S4 Table.** Distribution and 28-day mortality according to original SOFA score components.

**S5 Table.** Area under the receiver operating characteristic for predicting in-hospital mortality using the original cardiovascular/total SOFA and candidate models.

**S1 Fig.** Study population.

**S2 Fig.** Distribution and in-hospital mortality according to candidate cardiovascular/total SOFA scores.

**S3 Fig.** Calibration plots for in-hospital mortality of the candidate cardiovascular/total SOFA models.

**S1 Table. Conversion table of norepinephrine equivalent dose.**

| **Vasopressors** | **Dose** | **Norepinephrine equivalent** |
| --- | --- | --- |
| Epinephrine | 0.1 µg/kg/min | 0.1 µg/kg/min |
| Dopamine | 15 µg/kg/min | 0.1 µg/kg/min |
| Norepinephrine | 0.1 µg/kg/min | 0.1 µg/kg/min |
| Vasopressin | 0.04 U/min | 0.1 µg/kg/min |
| Phenylephrine | 1 µg/kg/min | 0.1 µg/kg/min |

**S2 Table. Candidate models for modified cardiovascular SOFA score with lactate criteria.**

| **Model** | **Score 0** | **Score 1** | **Score 2** | **Score 3** | **Score 4** | **Lactate criteria** |
| --- | --- | --- | --- | --- | --- | --- |
| **M1** | MAP ≥70 mmHg | MAP <70 mmHg *OR*  NEq ≤0.1 | 0.1< NEq ≤0.2 | NEq >0.2 | NEq >0.2 *AND*  Lactate ≥2 mmol/L | Add 1 point if lactate ≥2 mmol/L  from Score 0 to 3 |
| **M2** | MAP ≥70 mmHg | MAP <70 mmHg *OR*  NEq ≤0.1 | 0.1< NEq ≤0.2 | NEq >0.2 | NEq >0.2 *AND*  Lactate ≥4 mmol/L | Add 1 point if lactate ≥4 mmol/L  from Score 0 to 3 |
| **M3** | MAP ≥70 mmHg | MAP <70 mmHg *OR*  NEq ≤0.2 | 0.2< NEq ≤0.5 | NEq >0.5 | NEq >0.5 *AND*  Lactate ≥2 mmol/L | Add 1 point if lactate ≥2 mmol/L  from Score 0 to 3 |
| **M4** | MAP ≥70 mmHg | MAP <70 mmHg *OR*  NEq ≤0.2 | 0.2< NEq ≤0.5 | NEq >0.5 | NEq >0.5 *AND*  Lactate ≥4 mmol/L | Add 1 point if lactate ≥4 mmol/L  from Score 0 to 3 |
| **M5** | MAP ≥70 mmHg | MAP <70 mmHg *OR*  NEq ≤0.25 | 0.25< NEq ≤0.5 | NEq >0.5 | NEq >0.5 *AND*  Lactate ≥2 mmol/L | Add 1 point if lactate ≥2 mmol/L  from Score 0 to 3 |
| **M6** | MAP ≥70 mmHg | MAP <70 mmHg *OR*  NEq ≤0.25 | 0.25< NEq ≤0.5 | NEq >0.5 | NEq >0.5 *AND*  Lactate ≥4 mmol/L | Add 1 point if lactate ≥4 mmol/L  from Score 0 to 3 |
| **M7** | MAP ≥70 mmHg | MAP <70 mmHg *OR*  NEq ≤0.25 | 0.25< NEq ≤1.0 | NEq >1.0 | NEq >1.0 *AND*  Lactate ≥2 mmol/L | Add 1 point if lactate ≥2 mmol/L  from Score 0 to 3 |
| **M8** | MAP ≥70 mmHg | MAP <70 mmHg *OR*  NEq ≤0.25 | 0.25< NEq ≤1.0 | NEq >1.0 | NEq >1.0 *AND*  Lactate ≥4 mmol/L | Add 1 point if lactate ≥4 mmol/L  from Score 0 to 3 |
| **M9** | MAP ≥70 mmHg | MAP <70 mmHg | NEq ≤0.1 | 0.1< NEq ≤0.2 | NEq >0.2 | Add 1 point if lactate ≥2 mmol/L  from Score 0 to 3 |
| **M10** | MAP ≥70 mmHg | MAP <70 mmHg | NEq ≤0.1 | 0.1< NEq ≤0.2 | NEq >0.2 | Add 1 point if lactate ≥4 mmol/L  from Score 0 to 3 |
| **M11** | MAP ≥70 mmHg | MAP <70 mmHg | NEq ≤0.2 | 0.2< NEq ≤0.5 | NEq >0.5 | Add 1 point if lactate ≥2 mmol/L  from Score 0 to 3 |
| **M12** | MAP ≥70 mmHg | MAP <70 mmHg | NEq ≤0.2 | 0.2< NEq ≤0.5 | NEq >0.5 | Add 1 point if lactate ≥2 mmol/L  from Score 0 to 3 |
| **M13** | MAP ≥70 mmHg | MAP <70 mmHg | NEq ≤0.25 | 0.25< NEq ≤0.5 | NEq >0.5 | Add 1 point if lactate ≥2 mmol/L  from Score 0 to 3 |
| **M14** | MAP ≥70 mmHg | MAP <70 mmHg | NEq ≤0.25 | 0.25< NEq ≤0.5 | NEq >0.5 | Add 1 point if lactate ≥2 mmol/L  from Score 0 to 3 |
| **M15** | MAP ≥70 mmHg | MAP <70 mmHg | NEq ≤0.25 | 0.25< NEq ≤1.0 | NEq >0.5 | Add 1 point if lactate ≥2 mmol/L  from Score 0 to 3 |
| **M16** | MAP ≥70 mmHg | MAP <70 mmHg | NEq ≤0.25 | 0.25< NEq ≤1.0 | NEq >1.0 | Add 1 point if lactate ≥2 mmol/L  from Score 0 to 3 |

MAP = mean arterial pressure, NEq = norepinephrine equivalent dose (µg/kg/min).

Cut-off doses were selected by the norepinephrine dose of the original cardiovascular SOFA (0.1 µg/kg/min), the tertile of norepinephrine equivalent doses (0.1-0.2 µg/kg/min), the closest-to-(0,1) (0.2 µg/kg/min), the Youden index (0.25 µg/kg/min) in the derivation cohort of the original study, and “a priori” values (0.5 and 1.0 µg/kg/min).

**S3 Table. Candidate Models for Vasopressor Only Cardiovascular SOFA score.**

| **Model** | **Score 0** | **Score 1** | **Score 2** | **Score 3** | **Score 4** |
| --- | --- | --- | --- | --- | --- |
| V1 | MAP ≥70 mmHg | MAP <70 mmHg | NEq ≤0.1 | 0.1< NEq ≤0.2 | NEq >0.2 |
| V2 | MAP ≥70 mmHg | MAP <70 mmHg | NEq ≤0.2 | 0.2< NEq ≤0.5 | NEq >0.5 |
| V3 | MAP ≥70 mmHg | MAP <70 mmHg | NEq ≤0.1 | 0.1< NEq ≤0.25 | NEq >0.25 |
| V4 | MAP ≥70 mmHg | MAP <70 mmHg | NEq ≤0.25 | 0.25< NEq ≤0.5 | NEq >0.5 |
| V5 | MAP ≥70 mmHg | MAP <70 mmHg | NEq ≤0.2 | 0.2< NEq ≤1.0 | NEq >1.0 |
| V6 | MAP ≥70 mmHg | MAP <70 mmHg *OR*  NEq ≤0.1 | 0.1< NEq ≤0.2 | 0.2< NEq ≤0.5 | NEq >0.5 |
| V7 | MAP ≥70 mmHg | MAP <70 mmHg *OR*  NEq ≤0.1 | 0.1< NEq ≤0.25 | 0.25< NEq ≤0.5 | NEq >0.5 |
| V8 | MAP ≥70 mmHg | MAP <70 mmHg *OR*  NEq ≤0.1 | 0.1< NEq ≤0.5 | 0.5< NEq ≤1.0 | NEq >1.0 |
| V9 | MAP ≥70 mmHg | MAP <70 mmHg *OR*  NEq ≤0.25 | 0.25< NEq ≤0.5 | 0.5< NEq ≤1.0 | NEq >1.0 |
| V10 | MAP ≥70 mmHg | MAP <70 mmHg *OR*  NEq ≤0.2 | 0.2< NEq ≤0.5 | 0.5< NEq ≤1.0 | NEq >1.0 |
| V11 | No vasopressor use | NEq ≤0.1 | 0.1< NEq ≤0.2 | 0.2< NEq ≤0.5 | NEq >0.5 |
| V12 | No vasopressor use | NEq ≤0.1 | 0.1< NEq ≤0.5 | 0.5< NEq ≤1.0 | NEq >1.0 |
| V13 | No vasopressor use | NEq ≤0.25 | 0.25< NEq ≤0.5 | 0.5< NEq ≤1.0 | NEq >1.0 |
| V14 | No vasopressor use | NEq ≤0.2 | 0.2< NEq ≤0.5 | 0.5< NEq ≤1.0 | NEq >1.0 |
| V15 | MAP ≥70 mmHg | MAP <70 mmHg | Dopamine ≤5  Dobutamine (any dose) | Dopamine >5 OR  epinephrine ≤0.1 OR norepinephrine ≤0.1  OR NEq ≤0.1by vasopressin or phenylephrine dose | Dopamine >15 OR  epinephrine >0.1 OR norepinephrine >0.1  OR NEq >0.1 by vasopressin and phenylephrine dose |
| V16 | MAP ≥70 mmHg | MAP <70 mmHg | NEq ≤0.03 | 0.03< NEq ≤0.1 | NEq >0.1 |

MAP, mean arterial pressure; NEq, norepinephrine equivalent dose (µg/kg/min)

Cut-off doses were selected by the dopamine and norepinephrine dose of the original cardiovascular SOFA (0.03 and 0.1 µg/kg/min), the tertile of norepinephrine equivalent doses (0.1-0.2 µg/kg/min), the closest-to-(0,1) (0.2 µg/kg/min), the Youden index (0.25 µg/kg/min) in the derivation cohort of the original study, and “a priori” values (0.5 and 1.0 µg/kg/

**S4 Table. Distribution and in-hospital mortality according to original SOFA score components.**

| **Variables** | **Incidence, N (%)** | **In-hospital mortality, N (%)** |
| --- | --- | --- |
| Cardiac SOFA |  |  |
| 0 | 5,890 (19.9) | 437 (7.4) |
| 1 | 17,182 (58.0) | 1,507 (8.8) |
| 2 | 133 (0.5) | 33 (24.8) |
| 3 | 2,674 (9.0) | 313 (11.7) |
| 4 | 3,739 (12.6) | 1,385 (37.0) |
| CNS SOFA |  |  |
| 0 | 11,424 (38.6) | 1,027 (9.0) |
| 1 | 9,652 (32.6) | 624 (6.5) |
| 2 | 3,012 (10.2) | 446 (14.8) |
| 3 | 2,904 (9.8) | 635 (21.9) |
| 4 | 2,626 (8.9) | 943 (35.9) |
| Respiratory SOFA |  |  |
| 0 | 17,319 (58.5) | 1,573 (9.1) |
| 1 | 1,076 (3.6) | 106 (9.6) |
| 2 | 4,887 (16.5) | 594 (12.2) |
| 3 | 3,745 (12.6) | 567 (15.1) |
| 4 | 2,591 (8.8) | 835 (32.2) |
| Renal SOFA |  |  |
| 0 | 16,453 (55.6) | 1,043 (6.3) |
| 1 | 6,560 (22.2) | 786 (12.0) |
| 2 | 2,458 (8.3) | 476 (19.4) |
| 3 | 2,194 (7.4) | 627 (28.6) |
| 4 | 1,953 (6.6) | 743 (38.0) |
| Hepatic SOFA |  |  |
| 0 | 24,178 (81.6) | 2,351 (9.7) |
| 1 | 1,959 (6.6) | 375 (19.1) |
| 2 | 2,288 (7.7) | 511 (22.3) |
| 3 | 665 (2.3) | 196 (29.5) |
| 4 | 528 (1.8) | 242 (45.8) |
| Coagulation SOFA |  |  |
| 0 | 17,239 (58.2) | 1,892 (11.0) |
| 1 | 7,297 (24.6) | 700 (9.6) |
| 2 | 3,684 (12.4) | 603 (16.4) |
| 3 | 1,080 (3.7) | 348 (32.2) |
| 4 | 318 (1.1) | 132 (41.5) |

**S5 Table. Area under the receiver operating characteristic for predicting in-hospital mortality in the original and candidate models.**

| **Model** | AUROC of CV SOFA (95% CI) | p | AUROC of total SOFA (95% CI) | p |
| --- | --- | --- | --- | --- |
| **Original** | 0.663 (0.654-0.673) | **Reference** | 0.785 (0.777-0.793) | **Reference** |
| M1 | **0.675 (0.665-0.685)** | **<0.001** | 0.785 (0.777-0.793) | 0.733 |
| M2 | **0.684 (0.674-0.693)** | **<0.001** | **0.790 (0.782-0.798)** | **<0.001** |
| M3 | 0.667 (0.657-0.677) | 0.283 | 0.784 (0.776-0.793) | 0.490 |
| M4 | **0.676 (0.667-0.686)** | **<0.001** | **0.790 (0.782-0.798)** | **<0.001** |
| M5 | 0.664 (0.654-0.673) | 0.952 | 0.784 (0.776-0.793) | 0.482 |
| M6 | **0.673 (0.664-0.682)** | **0.004** | **0.790 (0.782-0.798)** | **<0.001** |
| M7 | 0.662 (0.652-0.672) | 0.745 | 0.783 (0.775-0.791) | 0.053 |
| M8 | **0.672 (0.663-0.681)** | **0.009** | **0.788 (0.78-0.796)** | **0.004** |
| M9 | 0.665 (0.655-0.675) | 0.549 | 0.779 (0.771-0.787) | <0.001 |
| M10 | **0.672 (0.662-0.682)** | **<0.001** | 0.785 (0.777-0.793) | 0.712 |
| M11 | 0.668 (0.658-0.678) | 0.102 | 0.780 (0.772-0.789) | <0.001 |
| M12 | **0.675 (0.665-0.685)** | **<0.001** | 0.786 (0.778-0.794) | 0.286 |
| M13 | 0.667 (0.658-0.677) | 0.146 | 0.781 (0.772-0.789) | <0.001 |
| M14 | **0.674 (0.665-0.684)** | **<0.001** | 0.786 (0.778-0.794) | 0.179 |
| M15 | 0.667 (0.657-0.677) | 0.176 | 0.781 (0.772-0.789) | <0.001 |
| M16 | **0.674 (0.664-0.684)** | **<0.001** | 0.786 (0.778-0.794) | 0.367 |
| V1 | 0.659 (0.649-0.669) | 0.068 | 0.784 (0.776-0.792) | 0.282 |
| V2 | 0.658 (0.648-0.668) | 0.027 | 0.785 (0.777-0.793) | 0.892 |
| V3 | 0.661 (0.651-0.671) | 0.284 | 0.785 (0.777-0.793) | 0.561 |
| V4 | 0.656 (0.647-0.666) | 0.002 | 0.785 (0.777-0.793) | 0.930 |
| V5 | 0.656 (0.646-0.665) | 0.001 | 0.784 (0.776-0.792) | 0.140 |
| V6 | 0.666 (0.656-0.675) | 0.330 | **0.790 (0.781-0.798)** | **<0.001** |
| V7 | 0.666 (0.656-0.675) | 0.296 | **0.790 (0.782-0.798)** | **<0.001** |
| V8 | 0.662 (0.652-0.671) | 0.497 | **0.787 (0.779-0.795)** | **0.040** |
| V9 | 0.642 (0.633-0.651) | <0.001 | **0.788 (0.779-0.796)** | **0.028** |
| V10 | 0.650 (0.641-0.659) | <0.001 | **0.788 (0.779-0.796)** | **0.022** |
| V11 | 0.655 (0.645-0.664) | 0.005 | **0.787 (0.779-0.795)** | **0.026** |
| V12 | 0.651 (0.641-0.660) | <0.001 | 0.786 (0.777-0.794) | 0.673 |
| V13 | 0.649 (0.639-0.658) | <0.001 | 0.787 (0.779-0.795) | 0.073 |
| V14 | 0.651 (0.641-0.660) | <0.001 | 0.787 (0.779-0.795) | 0.096 |
| V15 | 0.652 (0.642-0.662) | <0.001 | 0.777 (0.769-0.786) | <0.001 |
| V16 | 0.649 (0.639-0.659) | <0.001 | 0.785 (0.777-0.793) | <0.001 |

**S1 Fig. Study population.**

Adult patients with suspected infection who admitted to the ICU

(n=29,618)

Survival to hospital discharge

(n=25,943)

69,211 ICU admission episodes

16,061 repeated admission

23,532 patients without infection on the first day of ICU

In-hospital mortality (n=3,675)

**S2 Fig. Distribution and in-hospital mortality according to candidate cardiovascular/total SOFA scores.** Bar graphs represent the number of patients, and points with error bars indicate 28-day mortality with 95% confidence intervals. Abbreviations: SOFA, sequential organ failure assessment.

1. **modified Cardiovascular SOFA**


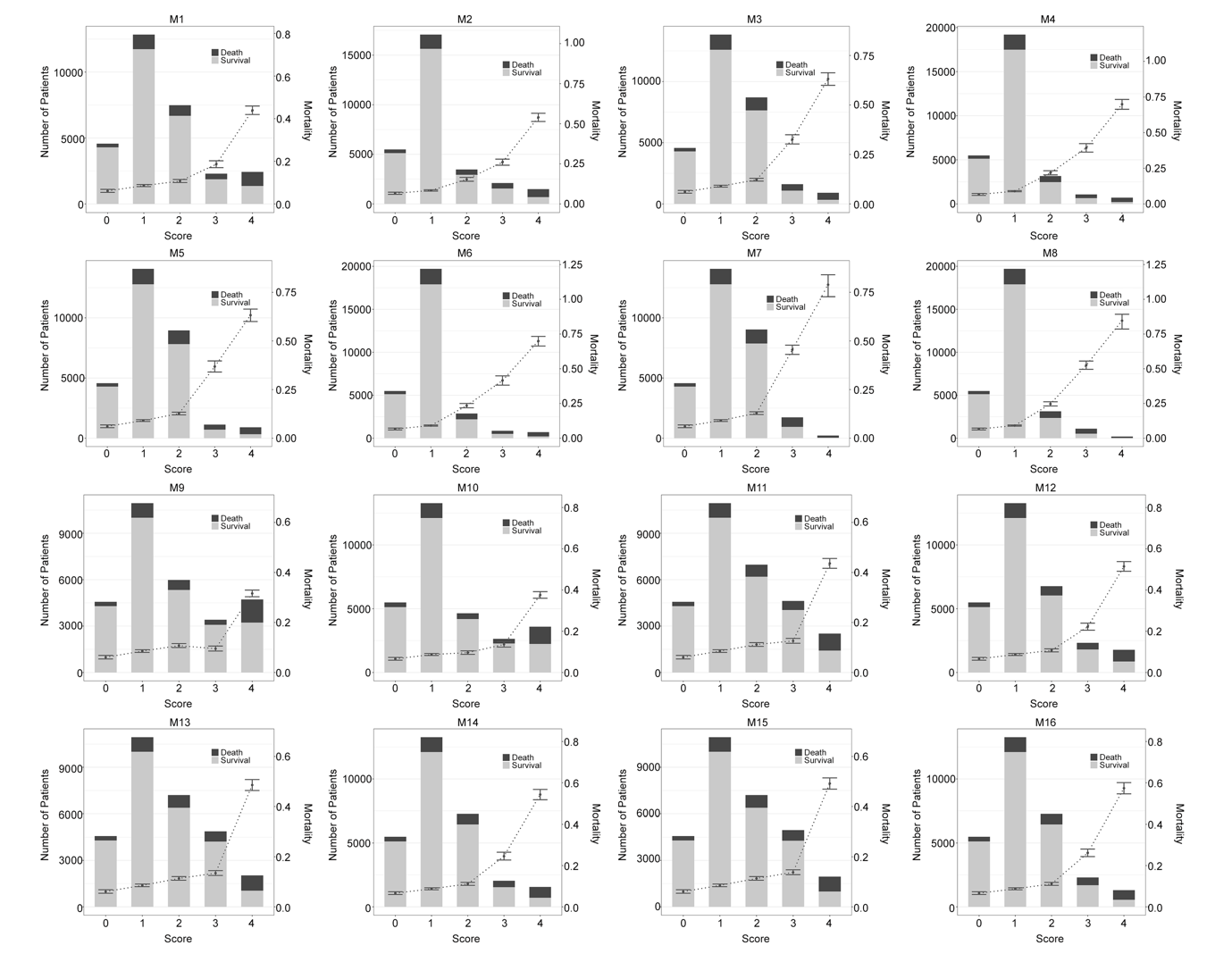


1. **vasopressor only CV SOFA**


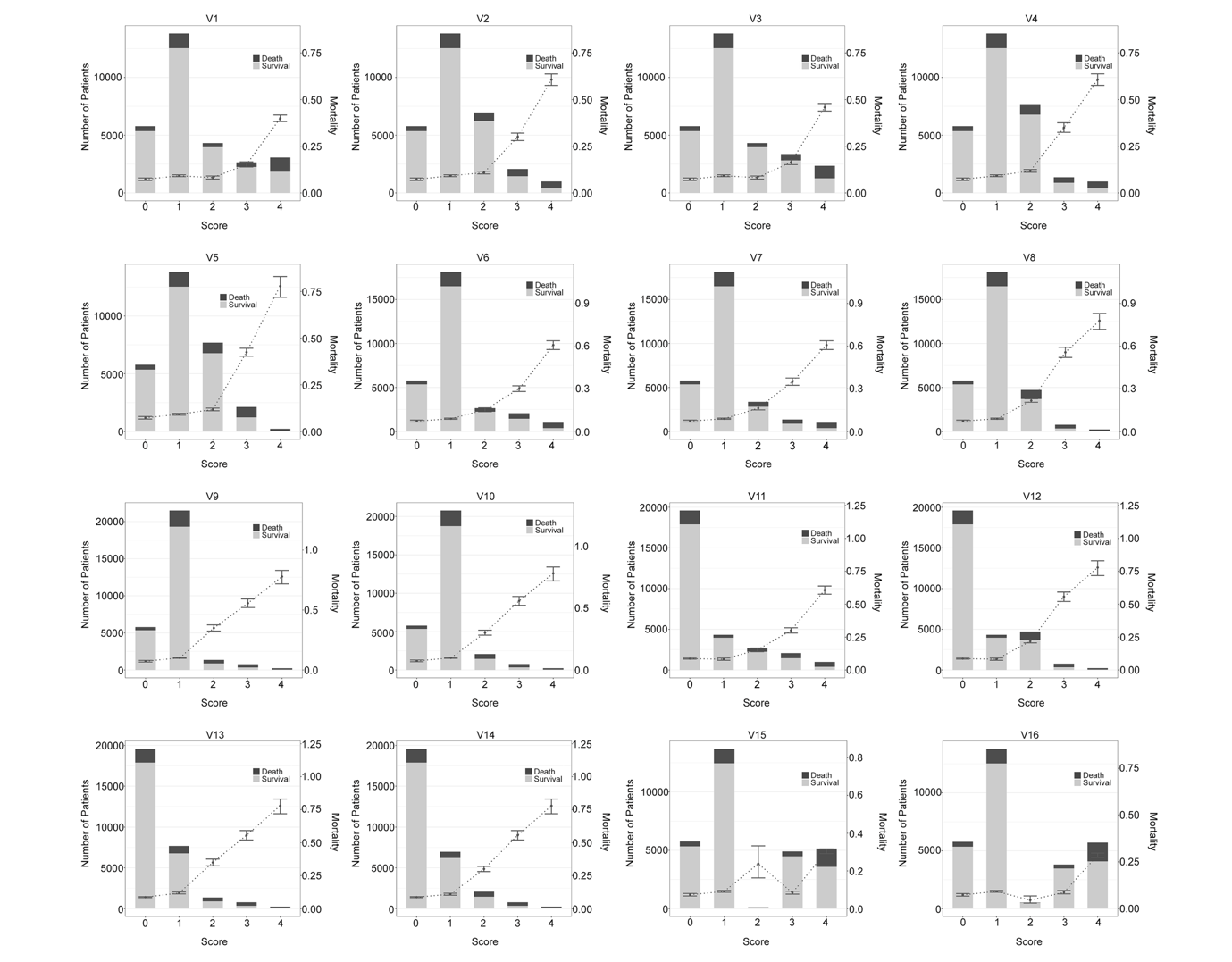


**S3 Fig. Calibration plots for in-hospital mortality of the candidate cardiovascular/total SOFA models.**

1. **modified Cardiovascular SOFA**


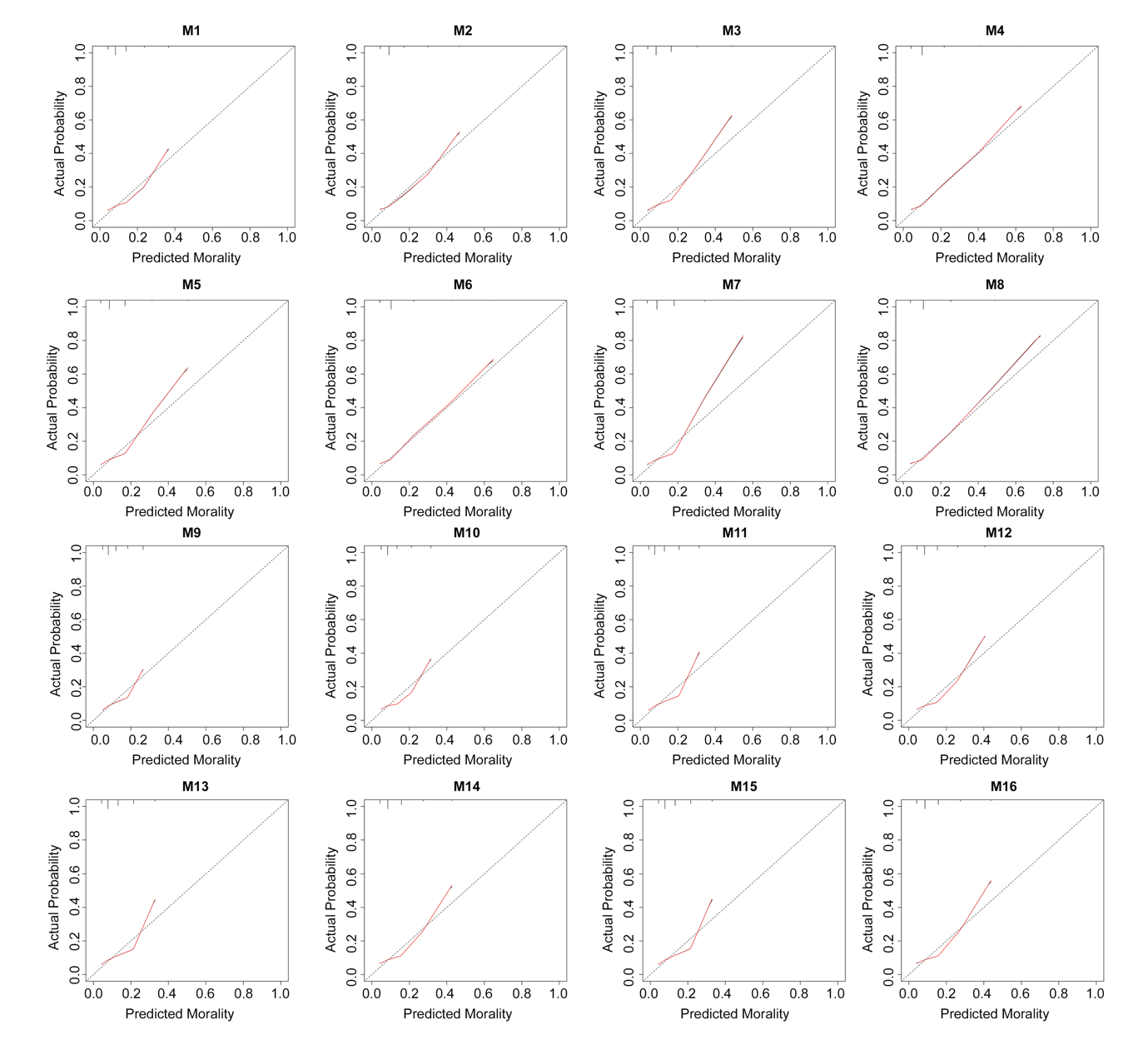


1. **vasopressor only CV SOFA**


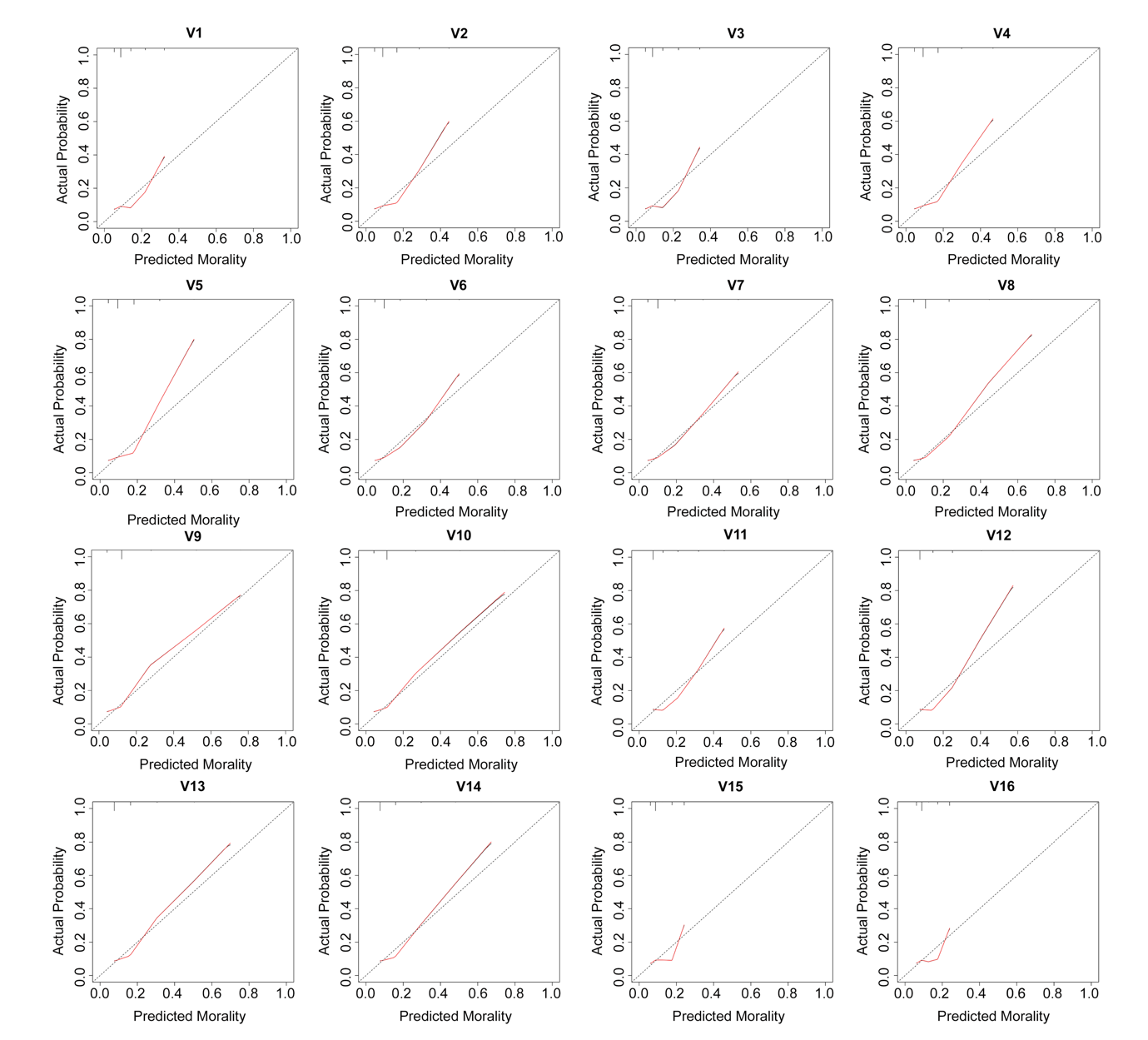

Supplement: S1 File — (DOCX) [file pone.0312185.s001.docx]
